# Synergistic Effects of Silicon and Preservative on Promoting Postharvest Performance of Cut Flowers of Peony (*Paeonia lactiflora* Pall.)

**DOI:** 10.3390/ijms232113211

**Published:** 2022-10-30

**Authors:** Jinnan Song, Jingli Yang, Byoung Ryong Jeong

**Affiliations:** 1 Department of Horticulture, Division of Applied Life Science (BK21 Four Program), Graduate School of Gyeongsang National University, Jinju 52828, Korea; 2 Institute of Agriculture and Life Science, Gyeongsang National University, Jinju 52828, Korea; 3 Research Institute of Life Science, Gyeongsang National University, Jinju 52828, Korea

**Keywords:** antioxidative capacity, carbohydrate stock, flower opening stage, holding (vase) solution, lipid peroxidation, reactive oxygen species (ROS), vase life

## Abstract

As a commercial high-grade cut flower, the marketability of herbaceous peony (*Paeonia lactiflora* Pall.) is limited by its short vase life in water. Si (silicon) is an alternative to improve the postharvest life of cut flowers. However, the effects of the combined application of Si and preservatives on the postharvest performance of cut peony flowers are unknown. In this study, the effects of a Si application and a preservative alone and collegial on the longevity of the vase life, water loss, antioxidant defense system, and stock carbohydrates level of cut flowers of three peony cultivars were investigated. It was observed that Si effectively prolonged the vase life, while the preservative alone, to a lesser extent, but markedly induced an early flowering and a greater flower diameter (flower open degree). The simultaneous use of Si and the preservatives not only showed larger flowers, but also improved the postharvest performance as characterized by an extended vase life and delayed the water loss. In addition, the Si supplementation dramatically intensified the antioxidant defense system (ameliorated antioxidant enzymes and alleviated ROS accumulation) in petals but did not increase the stock carbohydrates (starch and soluble sugars) levels, as compared to the treatment with the preservative alone. We show that a Si supplementation to a preservative is highly recommended for a large-scale use to promote the postharvest performance and competitiveness of marketed cut flowers.

## 1. Introduction

Herbaceous peony (*Paeonia lactiflora* Pall.) is an important ornamental plant species that has been widely distributed and cultivated in more than 50 countries [1]. *P. lactiflora* has well-recognized flowers, featuring plump shapes and vibrant colors, and these excellent aesthetic attributes render it as one of the most popular decorated flowers in the current international flower market [2,3]. Meanwhile, given the lush and fragrant flowers as well as the diverse range of cultivars, *P. lactiflora* is emerging as a novel high-end cut flower [4]. There is a considerable market demand in Korea for peony, where cut peony flowers with higher prices are imported from abroad, such as from Australia, to deal with the off-season lack, even though certain cultivars have been developed for domestic commercial production [5,6]. More importantly, the flowering duration is relatively short (around 7 days in spring only) and the vase life is inevitably influenced, which bottlenecks the availability and flexibility of the cut peony flower market [1,7]. Thus, fetching a better postharvest performance of cut peony flowers could sustain the industrialization development.

Certain parameters could be directly used to evaluate the postharvest performance of cut flowers. On the one hand, an imbalance of the water status, as characterized by the reduced plant fresh weight, can cause physiological changes regarding dehydration and wilting [8]. On the other hand, these two factors involving the flower opening and senescence also determine the vase life [9]. Furthermore, the stress degree of cut flowers will be amplified after harvest, due to the inhibited transpiration and disturbed metabolism. These accumulated stresses could lead to an overproduction of ROS (reactive oxygen species) and induce the expression of antioxidant enzymes together with the overall consumption of the carbohydrate stock [10,11].

To prolong the vase life and improve the postharvest performance of cut flowers, several techniques and strategies have been experimented with varying degrees of success [12,13]. Among them, a desired storage environment with low temperatures and humidity supplied by large equipment is regarded as a feasible guideline; however, it is costly and ineffective. Accordingly, the modification of the ingredients of the holding solution (vase solution) is still the most prominent method because of the easy and inexpensive operations [8,14]. With respect to the large-scale utilization of additional chemical agents in the holding solution, a non-toxic and stable preservative is highly recommended [1,11,15].

The use of silicon (Si) fertilization is one of these attempts that successfully extends the vase life of cut flowers, even though it is not recognized as a necessary element for higher plants [8,16]. Si has been suggested to reduce the transpiration rate by the formation of silica deposits on the leaf epidermis, which contributes to less water loss [17]. In a similar way, researchers indicated that the Si deposition was regarded as a mechanical barrier preventing fungal penetration, such as by *P. ultimum*, which has been shown to damage the water translocation system, resulting in damping off [18,19]. Moreover, many previous reports reveal that Si has a role in the mitigation of abiotic stresses through the modulation of the antioxidant system; for instance, the key antioxidant enzymes were boosted in Si-sufficient plants compared to their Si-deficient counterparts [8,20]. As a consequence, the detected accumulation of ROS was reduced accordingly. Furthermore, the Si-promoted antioxidant enzymes are reported to lower the membrane damage caused by MDA (malondialdehyde), which was identified as the end-product of lipid peroxidation [21,22].

Most commercial floral preservatives are manufactured by adding a sucrose and a biocide as the energy source and an inhibitor for bacterial growth, respectively [7,23]. However, these synthetic floral preservatives are free of Si according to Nguyen and Lim [15], because the knowledge of the positive impacts of Si on the postharvest performance of cut flowers remain incipient to date. Furthermore, most of the findings regarding the effects of Si on the postharvest performance are focused on the optimal forms of the Si source and how effective Si is against plant diseases [24,25,26,27]. Additionally, it is hypothesized that the effects of Si on the blooming and flower characteristics are markedly pronounced only when the plants are under stress conditions or with a long-term Si application [28], as illustrated in Sidi’s study [29]. Neither the comparative effects between Si alone nor the co-application with commercial floral preservatives have been investigated. In addition, Rabiza-Świder presents that the responses of the postharvest treatments are cultivar-specific, thereby the modifications of the holding solution should be individually tailored for a given cultivar [7].

Thus, modified holding solutions that can more effectively prolong the vase life and concomitantly enhance the plant quality should be developed. The objective of the present work is to: (1) assess and compare the effects of amended Si and/or a floral preservative in the vase solutions on the vase life and dynamic fresh weight loss of three cut peony flower cultivars, and (2) to elucidate the possible mechanism of Si involved in the postharvest performance by determining the quality of cut peony flowers.

## 2. Results

### 2.1. Vase Life and Diameters of Cut Peony Flowers as Affected by the Four Treatments

**Figure 1:**
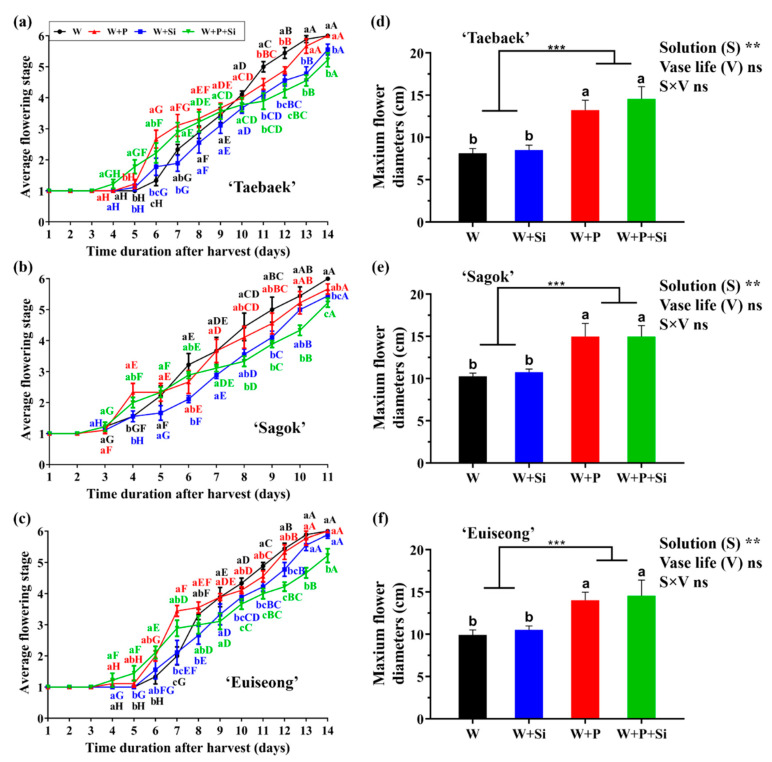
Effects of the four treatments on the vase life (**a**–**c**) and the maximum diameter (**d**–**f**) of cut peony flowers of three cultivars, namely ‘Taebaek’, ‘Sagok’, and ‘Euiseong’. Values are the average ± SE of *n* = 9 replicates. Different lowercase letters indicate the differences in flowering stages among treatments on the same day after harvest; different capital letters indicate the differences in flowering stages of same treatment on different days after harvest (one-way ANOVA with Duncan’s multiple comparison range test at *p* = 0.05); the significant differences between the P (−) and P (+) were determined following an unpaired two-tailed Student’s *t* test at *p* ˂ 0.001 (***), P(−) was treated as the control group; interactive effects of solution treatments and longevity of vase life on the maximum flower diameters are shown by ** (*p* ˂ 0.01) and ns determined by *F*-test. ‘W’: distilled water; ‘W + P’: distilled water supplemented with a preservative; ‘W+Si’: distilled water supplemented with silicon; ‘W + P + Si’: co-application of a preservative and silicon. Note that all the ‘W’, ‘W + P’, ‘W + Si’, and ‘W + P + Si’ appeared henceforth to carry the same meanings as mentioned here.

Either the preservative or Si significantly prolonged the vase life, regardless of the cultivar. Specifically, on day 12 during the vase life of ‘Taebaek’ and ‘Euiseong’, and on day 9 for ‘Sagok’, the cut flowers solely treated with water remarkably advanced about one flowering stage compared to the plants cultured in the other three groups (Figure 1a–c).

Concomitantly, more importantly, the co-application of the preservative and Si showed not only an early flowering (lowercase letters), but also a slow senescence speed (capital letters). As compared to the ‘W + P’, ‘W + P + Si’ of ‘Taebaek’, ‘Sagok’, and ‘Euiseong’ from day 10, day 8, and day 10 onwards, respectively, notably decreased the flowering stages. We then observed that the vase life shaped by Si was distinctly different from that shaped by the preservative. Before day 9, day 7, and day 8 during the vase life of ‘Taebaek’, ‘Sagok’, and ‘Euiseong’, respectively, the application of preservative led to an earlier flower opening. However, the cut flowers in the ‘W’ and ‘W + P’ groups displayed a faster flower opening after the days mentioned above, irrespective of the cultivar. By contrast, as expected, the cut stems cultured in the Si-sufficient holding solutions possessed a significantly extended vase life in comparison to the Si-deficient counterparts, particularly after the days mentioned above.

In addition, regardless of the cultivar, an addition of the preservative to the holding solutions significantly improved the flower diameters, whereas neither the supplementations of Si to ‘W’ nor to ‘W + P’ led to an increase in the flower diameter (no statistical differences), as displayed in Figure 1d–f. Additionally, no significant (ns) interaction was conferred between the treatment solutions and longevity of the vase life on the maximum flower diameter.

### 2.2. Fresh Weight Loss of Cut Stems during the Vase Life as Affected by the Four Treatments

**Figure 2:**
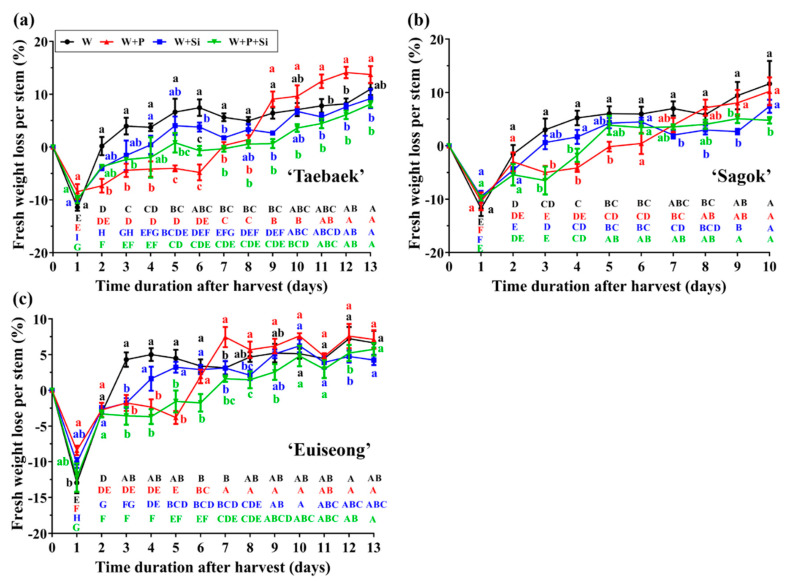
Effects of the four treatments on the fresh weight loss of the three cultivars, namely (**a**) ‘Taebaek’, (**b**) ‘Sagok’, and (**c**) ‘Euiseong’. Data presented are the mean ± SE of *n* = 9 replicates. Different lowercase letters indicate the differences in fresh weight lose among treatments on the same day after harvest; different capital letters indicate the differences in fresh weight loss of same treatment on different days after harvest (one-way ANOVA with Duncan’s multiple comparison range test at *p* = 0.05).

The cut stems firstly absorbed varying amounts of water when the vase life started, and gradual water losses were monitored afterwards, regardless of the cultivar and treatment (Figure 2). It is noteworthy that the cut stems of the three cultivars treated in the ‘W’ groups showed a more rapid water uptake and water loss rates (−10 to 5% approximately) during the first three days of their vase life.

Furthermore, the water uptake or water loss rate varied greatly among the treatments considered. Regarding the cut stems treated without the preservative, the water loss of the cut flowers in the ‘W’ group was observed from day 2, while the cut flowers held in ‘W + Si’ started to lose water on day 4, day 3, and day 3, respectively, in ‘Taebaek’ (Figure 2a), ‘Sagok’ (Figure 2b), and ‘Euiseong’ (Figure 2c). Still, the cut stems cultivated with the preservative in the holding solutions exhibited a relatively delayed water loss. For example, the water loss of cut stems in ‘Taebaek’ (Figure 2a), ‘Sagok’ (Figure 2b), and ‘Euiseong’ (Figure 2c) in the ‘W + P + Si’ group started on day 5, day 4, and day 6, respectively.

Between the Si-deficient and Si-sufficient holding solutions, the cut peony flowers nourished with Si had a significantly lower water loss rate (lowercase letters) together with a more stable water status (capital letters), especially in the middle and near the end of the vase life (Figure 2). In particular, from day 8, day 8, and day 6 onwards, respectively, of ‘Taebaek’, ‘Sagok’, and ‘Euiseong’, the ‘W + P + Si’ group saw a significant decline in the fresh weight loss rate compared to that in the ‘W + P’ group.

### 2.3. Effects of the Four Treatments on the Major Antioxidant Enzyme Activities

**Figure 3:**
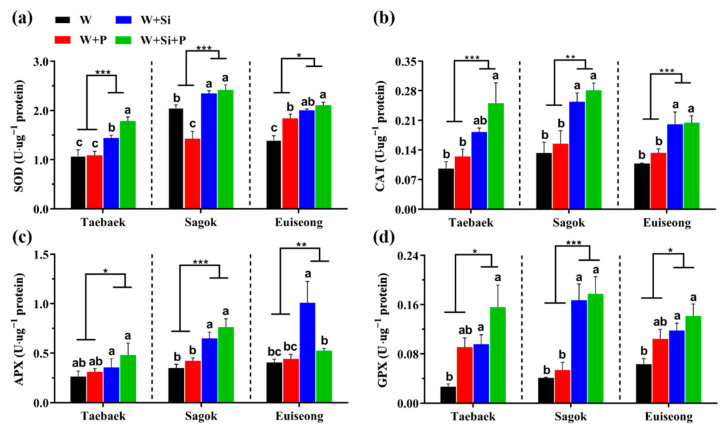
Activities of key antioxidant enzymes: (**a**) superoxide dismutase (SOD), (**b**) catalase (CAT), (**c**) ascorbate peroxidase (APX), and (**d**) guaiacol peroxidase (GPX) in cut peony petals of ‘Taebaek’, ‘Sagok’, and ‘Euiseong’ as affected by the four treatments. Data are the average ± SE generated from *n* = 4 technological replicates. Statistical differences among four treatments were determined by Duncan’s multiple comparison range test at *p* = 0.05 and denoted by different lowercase letters. The significant differences between the Si (−) and Si (+) were determined following an unpaired two-tailed Student’s *t* test at *p* ˂ 0.05 (*), ˂ 0.01 (**), and ˂ 0.001 (***), Si (−) was regarded as the control group.

The results pertaining to the major antioxidant enzyme activities detected in the petals are shown in Figure 3. Outstandingly, Si additions to either the ‘W’ or ‘W + P’ vase solutions remarkably enhanced these four major antioxidant enzyme concentrations, regardless of the cultivar. For instance, the cut peony flowers of ‘Taebaek’ cultivated in the ‘W + Si’ vase solutions significantly possessed a 34.6% and 33.3% enhancement of SOD activity, respectively, relative to those cultivated in ‘W’ and ‘W + P’ (Figure 3a); similarly in ‘Taebaek’, a Si supplementation to the ‘W + P’ group (‘W + Si + P’) dramatically improved the SOD activity by 67.3% and 65.7% compared to the ‘W’ and ‘W+P’ groups, respectively (Figure 3a). Similar trends can be found in the results regarding the other three antioxidant enzymes and the two cultivars (Figure 3b–d).

In most cases, the addition of the preservative to the ‘W’ solutions failed to improve the activity of the key antioxidant enzymes (no statistical differences); the SOD activity in the ‘Sagok’ petals cultivated in ‘W + P’ even decreased by 30.7% as compared to that in the ‘W’ group (Figure 3a). Meanwhile, in comparison with the cut peony flowers from ‘W + Si’, the added preservative did not show a statistical difference in the antioxidant enzyme activity, either, such as that of APX and GPX (Figure 3c,d).

### 2.4. Effects of the Four Treatments on the ROS (Reactive Oxygen Species) Accumulation

**Figure 4:**
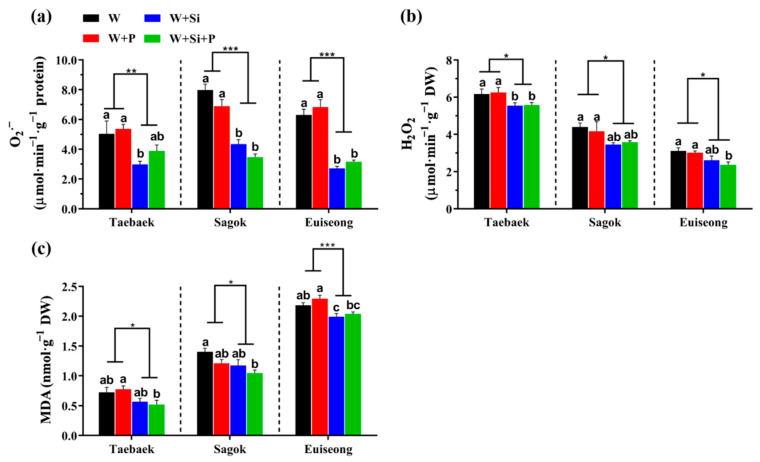
ROS accumulations in terms of (**a**) superoxide radical (O_2_^−^), (**b**) hydrogen peroxide (H_2_O_2_), and (**c**) malondialdehyde (MDA) contents in the petals of three peony cultivars as affected by the four treatments. Data are the average ± SE generated from *n* = 4 technical replicates. Statistical differences among the four treatments were determined by Duncan’s multiple comparison range test at *p* = 0.05 and indicated by different lowercase letters. The significant differences between the Si (−) and Si (+) were determined following an unpaired two-tailed Student’s t test at *p* ˂ 0.05 (*), ˂ 0.01 (**), and ˂ 0.001 (***), and Si (−) was regarded as the control group.

Consistent with the data obtained above, the improved antioxidant activities following a Si application significantly decreased the ROS concentrations in the petals, regardless of the cultivars (Figure 4). Notably, the cut peony flower petals of ‘Euiseong’ with a sole Si nutrition showed rapid diminishments in the O_2_^.-^ content by 60.0% and 60.31%, compared with that cultured in the ‘W’ and ‘W + P’ groups, respectively; also, the co-application of Si and the preservative (‘W + Si + P’) dramatically decreased the O_2_^.−^ content by 49.7% and 53.6%, respectively, relative to the ‘W’ group and ‘W + P’ group (Figure 4a). However, similar with the monitored antioxidant enzyme activities, statistical differences were not conferred in most cases by the supplementation of the preservative.

### 2.5. Responses of the Starch and Soluble Sugar Contents to the Four Treatments

**Figure 5:**
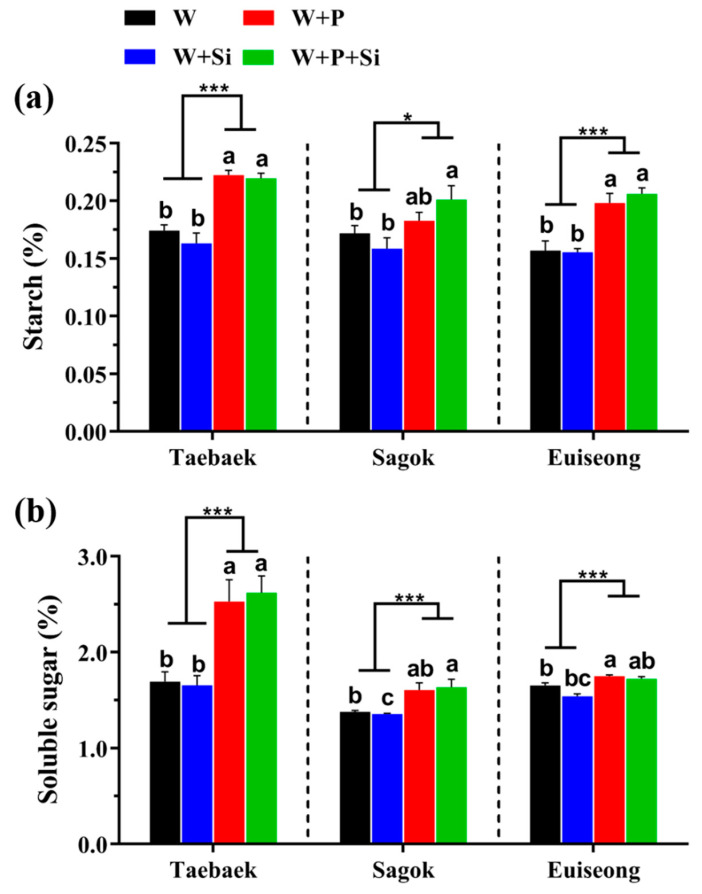
Carbohydrates stock in terms of (**a**) starch (%) and (**b**) soluble sugar (%) contents in the three cultivar petals as affected by the four treatments. *n* = 4 replicates were averaged and statically analyzed by following Duncan’s multiple comparison range test at *p* = 0.05 and revealed by different lowercase letters. Statistical differences between P (−) and P (+) were determined by an unpaired two-tailed Student’s t test at *p* ˂ 0.05 (*), and ˂ 0.001 (***), and P (−) was considered as the control group. Error bars denote means ± SE.

As is apparent in Figure 5, the added Si did not enhance the starch or soluble sugar content (no statistical differences and certain reduced tends were monitored) as compared to the Si-deficient petals, irrespective of the cultivar. On the contrary, additions of the commercial preservative to the holding solutions to varying degrees improved not only the starch concentration but also the soluble sugar content, independent of the Si concentration.

In addition, it is noteworthy that a sole Si addition to the holding solutions decreased both the starch and soluble sugar contents compared to the control, while the supplementations of Si to the ‘W + P’ groups significantly enhanced the carbohydrate stocks in ‘Sagok’ solely. However, no statistical differences were observed in ‘Taebaek’ and ‘Euiseong’ between the ‘W + P’ and ‘W + P + Si’ (Figure 5).

### 2.6. Differences in the Mechanisms Regarding Si and the Commercial Preservative Are Supported by the PCA

In order to visualize the effects of added Si and/or the preservative on the antioxidant defense system and carbohydrate stock, as well as to distinguish the mechanisms of applying Si and/or the preservative in promoting the postharvest performances, a PCA concerning the key antioxidant enzymes activities, ROS concentration, starch content, and soluble sugar level data set in combination with the four treatments and three cultivars, was performed.

**Figure 6:**
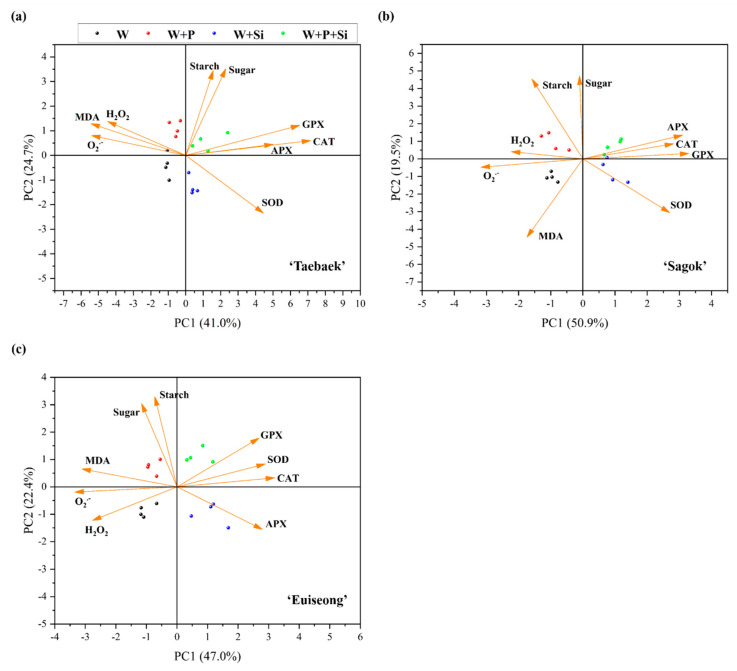
PCA (principal component analysis) on basis of the parameters related to the antioxidant defense system and carbohydrate stock in (**a**) ‘Taebaek’, (**b**) ‘Sagok’, and (**c**) ‘Euiseong’ subjected to the four treatments. SOD: superoxide dismutase, CAT: catalase, APX: ascorbate peroxidase, GPX: guaiacol peroxidase, O_2_^.−^: superoxide radical, H_2_O_2_: hydrogen peroxide, MDA: malondialdehyde.

The PCA results in ‘Taebaek’, ‘Sagok’, and ‘Euiseong’ showed that 65.7% (Figure 6a), 70.4% (Figure 6b), and 69.4% (Figure 6c), respectively, of the total data variability were declared of the first two principal components (PC1+PC2). On the whole, the Si-sufficient samples were distributed on the right side of the PC1 plot, whereas the Si-deficient samples were grouped on the left side of the PC1 plot (Figure 6 ‘PC1’). Similarly, the samples treated with the preservative were located on the upper quadrants of the PC2 plot, while the samples without the added preservative were separated on the lower quadrants of the PC2 plot (Figure 6 ‘PC2’).

Moreover, the Si-treated samples displayed positive impacts on the antioxidant enzyme activities, while they were negatively correlated with the ROS concentration, but these parameters could be not related to the starch and sugar contents (orthogonal).

## 3. Discussion

The flower commodity value is directly determined by the cut flower’s vase life [1]. Intuitively, the modification of the holding solution is a highly recommended way to delay the senescence and keep the freshness for a longer period [30]. Indeed, bulks of exploratory work regarding the selection of preservatives have been carried out for a better postharvest performance of cut flowers, such as nano-silver [1], 8-hydroxyquinoline sulfate [31], sugar [32], etc. However, on the one hand, many of them are deleterious to humans, which is not suitable for a large-scale application [33]; on the other hand, some of them are already contained in commercial preservatives that show a low efficiency and a greater susceptibility to microbial occlusion because of the presence of sugars [34]. Si is an eco-friendly and safe agent in cut flower preservation and has been reported to successfully extend the vase life and enhance the flower quality in carnation [35], Argyranthemum [36], and rose [11]. Unfortunately, few studies have focused on the co-application of Si and other preservatives, especially a standard commercial preservative [37]. Therefore, the study described herein aims to assess how Si and a commercial preservative synergistically influence the postharvest performance of cut peony and determine the differences in the mechanisms between them. The postharvest performances of cut peony flowers in this study were evaluated through the length of their vase life and water loss characteristics.

In our trail, the postharvest cut flower stages of three cultivars were distinctly separated into six periods based on the status and appearances of the flowers (Section 4.3), and these stages can be used as an indicator of the vase life and plant senescence.

### 3.1. Combined Use of Si and a Preservative Promotes Early Flowering but Prolongs the Vase Life

We observed that a Si application to cut stems showed delayed flower stages and a prolonged vase life (Figure 1a–c). Specifically, sole Si nutrition presented in the holding solutions had relatively retarded flower stages throughout the whole vase life as compared to the control and ‘W + P’; while the co-applied Si and a preservative to the holding solution displayed early flowering first and subsequently delayed the flower stages. This indicated that a postharvest Si usage can increase the vase life of cut peony flowers; these data were in contract with many previous reports [8,35,38,39]. This enhancement on the postharvest life with a Si application is probably due to the fact that the incorporation of Si into plant tissues can control the stomatal conductance and concomitantly well regulate the water status by reducing the transpiration rate [40,41].

By contrast, the cut stems cultivated with the supplemented commercial preservative possessed not only the accelerated flowering, but also the enhanced flower diameter (Figure 1d–f). However, the cut stems treated solely with the preservative (‘W + P’) displayed a relatively short vase life (about 1.5 flowering stages ahead) and were more prone to senescence compared to the Si-sufficient plants. Most likely, the composition of sugars presented in the commercial preservative was required as the energy for the bud opening and participation in the carbon skeleton of the floral structures [42,43]. Conversely, the presence of sugars in the holding solutions could cause phytotoxic reactions, as characterized by petal scorch and necrotic lesions [44]. 

As expected, when compared with the cut flowers grown in water only, the co-application of Si and the preservative in this study supplemented to the holding solution not only promoted an early flowering (about 3 days earlier for flowering) with larger flowers in diameter (about a 50% increase in the flower diameter), but also extended the postharvest life (about 1.5 flowering stages delayed before wilting) (Figure 1). These results suggested that the combined use of Si and the preservative was a more effective way to induce an early flowering and extend the vase life, rather than by a sole application.

### 3.2. Si Application Reduces Water Loss and Delays the Senescence of Cut Stems

Likewise, in addition to the flowering stages, the water status concerning the water uptake and loss is considered another critical indicator of the postharvest performance [8,45]. The daily fresh weight loss was used in this study to mirror the water status. During the ageing process, the membrane permeability can upsurge, resulting in the enhancement of water loss in petals. Therefore, decreasing the water loss or maintaining the water balance can assist in the inhibition of cut flower aging.

In our study, Si-deprived plants showed a more rapid water uptake and loss rate than the Si-sufficient counterparts, which presented a poor ability in maintaining the water balance by the former (Figure 2). Indeed, the formation of phytoliths by Si deposited at the stomata have been regarded as the physical barrier to coordinate the water loss by transpiration [46]. In addition, as Jaiganesh noted, the participation of Si was found to limit bacterial development, which was believed to contribute to a better water balance [47]. Meanwhile, the cut stems treated without Si (‘W’, ‘W + P’) exhibited a similar tendency in the dynamic water loss ratio after the fresh weight loss (Figure 2). Succinctly, a Si application reduces the water loss and delays the senescence of cut peony stems.

### 3.3. Si Application Intensified the Antioxidant Defense System but Did Not Significantly Increase the Carbohydrate Content

Apart from the morphological changes, a various biophysical and biochemical deterioration could be rendered during the petal aging [48]. All the detected changes were more pronounced near the end of the postharvest life [1,49]. The balance between the production and detoxification of ROS was damaged when the plants were under abiotic stresses [46]. The accumulation of ROS (O_2_^.−^, H_2_O_2_) stimulated the damage from the lipid peroxidation. Moreover, during the cut flower senescence, lipid peroxidation increased in the ageing petals, leading to a decline in the membrane integrity. Higher plants have evolved sophisticated mechanisms to scavenge the overproduction of ROS, such as increasing the antioxidant enzyme concentrations [50]. The ROS damage and oxidative stress degree are signposted by MDA, which is defined as the final product of lipid peroxidation [51]. The benefits of Si on the physiological and morphological aspects of plants have been extensively witnessed [16,52]; concurrently, Si inflicted the increase in the antioxidant production which was monitored to counteract the ROS accumulation [53].

Consistently, the Si augmentation considerably promoted the concentration of the antioxidant enzymes regarding SOD, CAT, APX, and GPX, regardless of the cultivar (Figure 3), which well agrees with previous reports in support of the beneficial role of Si on the antioxidant defense machinery [20,46,54,55]. Afterwards, the antioxidant enzymes triggered by Si meritoriously assisted the abrupt diminishment of the ROS contents including O_2_^.−^, H_2_O_2_, and MDA (Figure 4). It could be assumed that the delayed senescence and prolonged postharvest life of cut peony flowers stemmed from the enhanced antioxidant defense system by Si. Similar results in rose [11], tuberose [56], and muskmelon [57] also advocated the alleviatory effects of Si on the ROS damage. These results may explain why the Si-deficient cut stems had a relatively short vase life and poor postharvest performance.

Furthermore, certain carbohydrates (starch and sugar) are indispensable substrates for maintaining the postharvest performance. Structurally, during the flowering process, substantial amounts of carbohydrates are required for the bud opening as the carbon skeleton for the cell wall synthesis [42]. Biochemically, sugars were documented to enhance the osmotic pressure, suppress the sensitivity to ethylene, and supply energy for the cellar metabolism and physiological activities [58,59]. In our study, the cut stems treated with the commercial preservative were detected to have not only a significant larger flower diameter (Figure 1d–f), but also remarkably higher starch and soluble sugar contents as compared to those cultivated without the commercial preservative, regardless of the cultivars tested and Si supplementation (Figure 5). Concurrently, the PCA data elucidated that the improvements in the starch and sugar contents had little correlation with the presence of Si in the vase solutions (Figure 6).

However, the abundant carbon sources, especially in the vase solutions, are readily associated with the decreases in the cut stem water conductivity and increases in the bacterial growth, resulting in the vascular occlusion and, accordingly, the restricted vase life [60]. Consequently, the cut stems treated solely with the preservative (‘W + P’) showed a relatively unstable water status and a short vase life (Figure 1 and Figure 2). Thus, the promoted postharvest performance by Si may be ascribed to the intensified antioxidant defense system rather than increasing the carbohydrate contents (Figure 5 and Figure 6).

Si-treated cut peony flowers failed to improve the carbohydrate contents, probably due to the following: (1) the period of the Si application in this experiment was relatively short (only conducted during the postharvest period), where, accordingly, only small amounts of Si were taken up and integrated, thereby the promotions of Si on photosynthesis were less pronounced. (2) Energy is required for the transport of Si, which may cause the depletion in the carbohydrate reserves. (3) A Si-promoted hydraulic movement on the uptake of other beneficial ions is unavailable in this vase experiment [46,61].

## 4. Materials and Methods

### 4.1. Plant Materials and Growth Conditions

**Figure 7:**
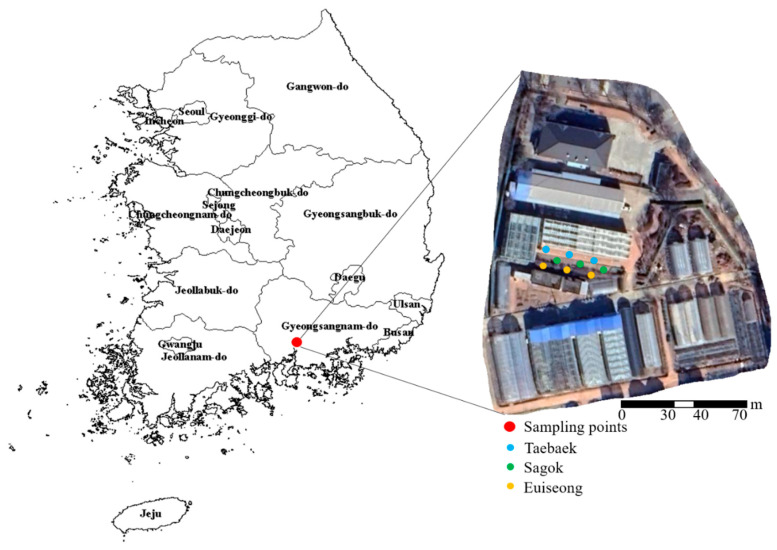
Location of the sampling site and cultivation area of the peony plants.

The city of Jinju is in the south part of the Republic of Korea (Figure 7) and has a warm temperate climate with an annual mean temperature from 10.5 to 18.2 °C [62]. The mean annual sunshine duration is 2210.4 h [62]. The mean annual precipitation is 1430 mm [62]. This study was carried out three times by using three herbaceous peony (*Paeonia lactiflora* Pall.) cultivars (‘Taebaek’, ‘Sagok’, and ‘Euiseong’) which were grown at Gyeongsang National University (35°81′ N, 128°01′ E, Jinju, Korea) under filed conditions for three successive years, from 2020 to 2022. The spacing of the sampling plots for identical cultivars is 10 meters, while for different cultivars it is 5 m (Figure 7).

When the peony plants formed a flower bud or entered the ‘marble like’ stage (in the end of April, with a temperature of 20 °C approximately), the stems with a uniform morphology but without any visible symptoms of disease, incorporated insects, or mechanical flaws were cut and trimmed to 40 cm [8,63]. The cut stems were processed by stripping off all but the four uppermost leaves which remained and immediately immersing them upright into beakers with distilled water. To provide an equal water uptake, all the obtained stems were transported to the lab in 10 minutes and subjected to a crosswise cut at the bottom to 30 cm in distilled water [1]. The lab temperature is about 25 °C without artificial lighting.

### 4.2. Treatments and Experimental Design

Subsequently, the stems were transferred to the vase solutions and placed in a controlled environment (uninterrupted white LED light at 300 PPFD and a constant temperature at 21 °C), provided by a refrigerated showcase (Refrigeration + LLC, Colorado Springs, CO, USA). The vase solutions designed in this study consisted of four treatments: distilled water ‘W’ (the control), distilled water supplemented with a standard preservative (a commercial floral preservative) prepared following the manufacturer’s manual (HydraFlor 100, FlorBelle, Oasis Corp, Seoul, Korea) ‘W + P’, distilled water supplemented with optimized Si sourced from a pure Si solution at 75 mg·L^−1^ [8,16] ‘W + Si’, and the co-applications of the commercial floral preservative and Si solubilized in distilled water ‘W + P + Si’. The main ingredients of the commercial floral preservative are glucose and citric acid, and it is free of Si [64]. Five hundred mL per vase of the treatment solution was initially added and replenished if required. For each treatment, studies were conducted in a completely randomized design by adopting three biological replicates. Each replicate contains three stems of the same cultivar, individually labeled, and inserted into the treatment solutions with one vase.

### 4.3. Flower Opening Stage Definitions and Observations

**Figure 8:**
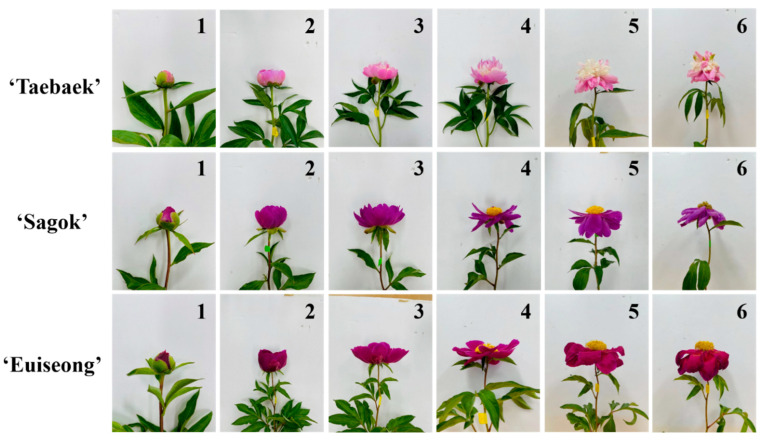
Six postharvest stages of cut peony flowers ‘Taebaek’, ‘Sagok’, and ‘Euiseong’. 1, ‘Marble-like stage’ or ‘Opening-engaged stage’. 2, ‘Initial-opening stage’. 3, ‘Half-opening stage’. 4, ‘Full-opening stage’. 5, ‘Petal-wilting stage’. 6, ‘Petal-falling stage’ or ‘Pistil-wilting stage’.

The postharvest stages of the cut peony flowers were immediately recorded after they were placed in the vase solutions. The flower opening stages during the flowers’ vase life can be distinctly divided into six phases according to the appearance and status (Figure 8). The plants developed a soft flower bud which was considered as the pre-opening stage (Stage 1). The petals can be readily observed while the pistils are detected solely from the top view, which is defined as the ‘Initial-opening stage’ (Stage 2). Half or nearly half of the flower opening is the key marker of Stage 3. Stages 4 is characterized by a complete opening of the flower. Flowers at the wilting stages (Stage 5 and Stage 6) are characterized by the petals and/or pistils rolling up or dropping together, with further decaying. The flower stages of the cut stems were individually observed and recorded daily until the last day of their vase life.

### 4.4. Measurement of the Daily Fresh Weight Loss

The cut stems were individually picked out of the vase solution, surface blotted with disposable wipers (Kimwipes, Sigma-Aldrich), and weighed using an electronic balance. The fresh weight loss ratio (%) was calculated as follows:

$$\mathrm{Fresh}\;\mathrm{weight}\;\mathrm{loss}\;\mathrm{per}\;\mathrm{stem}\;\left(\%\right)=\;\frac{\mathrm{the}\;\mathrm{fresh}\;\mathrm{weight}\;\mathrm{of}\;\mathrm{cut}\;\mathrm{stem}\;\mathrm{in}\;\mathrm{Day}\;\left(n+1\right)-\mathrm{the}\;\mathrm{fresh}\;\mathrm{weight}\;\mathrm{of}\;\mathrm{cut}\;\mathrm{stem}\;\mathrm{in}\;\mathrm{Day}\;\left(n\right)}{\mathrm{the}\;\mathrm{fresh}\;\mathrm{weight}\;\mathrm{of}\;\mathrm{cut}\;\mathrm{stem}\;\mathrm{in}\;\mathrm{Day}\;\left(n\right)}\times 100\%$$

### 4.5. Assays of the Key Antioxidant Enzyme Activities

The oxidative stress degree in the cut peony flowers was determined when they entered Stage 6. The fallen petals were collected, quickly immersed in liquid N_2_, and stored in a −81 °C freezer until the further experiment.

The activity of certain key antioxidant enzymes, namely, superoxide dismutase (SOD), catalase (CAT), ascorbate peroxidase (APX), and guaiacol peroxidase (GPX), were measured based on the extracted protein content. Specifically, the frozen samples were finely ground in mortars over an ice bath. Then, one hundred milligrams of the fine powder were homogenized in an extraction buffer containing 50 mM of PBS, 2% polyvinylpyrolidone, 1 mM of EDTA, and 0.05% triton-x, and adjusted so the pH value = 7.0 by adding 0.1 N of HCl. The supernatant was collected to new tubes after a centrifugation (13,000 rpm, 20 min, 4 °C). The total soluble protein content was quantified using the Braford reagent [65].

The SOD activity was determined by using the NBT (nitroblue tetrazolium) reduction according to Giannopolitis and Ries [66]. The CAT activity was monitored on basis of the decomposition of H_2_O_2_ [67]. The APX activity was assayed by adopting the ascorbate oxidation amount in the extracted protein samples [68]. A guaiacol oxidation reaction was employed for the estimation of GPX [69]. The detailed procedure was shown in Zhao’s publication [70].

### 4.6. Quantification of Superoxide Radical (O_2_^.−^), Hydrogen Peroxide (H_2_O_2_), and Malondialdehyde (MDA)

The oxidative stress markers including O_2_^.−^, H_2_O_2_, and MDA were spectrophotometrically quantified with a spectrophotometer (Libra S22 type, Biochrom, Cambridge, UK). Specifically, the O_2_^.−^ content was determined by following a method described by Wu, adopting the hydroxylamine oxidization strategy [71]. The H_2_O_2_ level was colorimetrically measured according to an approach proposed by Mukherjee [72]. The lipid peroxidation degree is signposted by the MDA concentration, which was assayed based on the TBA (thiobarbituric acid) reaction [73]. The specific protocol can be found in Li’s report [74].

### 4.7. Determination of the Carbohydrate Level

The carbohydrate level was determined herein in terms of the contents of starch and soluble sugars. A modified anthrone–sulfuric acid colorimetry method was used to quantify the two forms of carbohydrates in the petals [75]. Briefly, 0.3 grams of the fine petal powder was vigorously mixed with 25 mL of deionized water and extracted in a boiling water bath for at least 40 minutes. The mixture was then centrifuged at 6500 rpm for 10 min at RT after cooling down for 15 min, to acquire the supernatant for the soluble sugar assay. The soluble sugar content was determined in a reaction medium consisting of 0.2 mL of four times-diluted supernatant, 0.5 mL of 2% anthrone (daily prepared in darkness), and 1.8 mL of distilled water. The reaction was triggered with the addition of 5 mL of concentrated H_2_SO_4_ (sulfuric acid). The absorbance of the mixture solution was recorded spectrophotometrically at 630 nm.

The residue was homogenized afterwards with 2 mL of 9.6 M HClO_4_ (perchloric acid) and adjusted to 20 mL with deionized water, then the mixture was incubated in a water bath at 100 °C for 40 min. After a centrifugation (6500 rpm, 10 min, RT), 0.5 mL of the supernatant was mixed with 1.5 mL of distilled water and 1 mL of 2% anthrone (daily prepared in darkness). The reaction was started by the addition of 5 mL of concentrated H_2_SO_4_ (sulfuric acid). The absorbance of the mixture was spectrophotometrically read at 485 nm [76].

### 4.8. Statistics and Graph

All the exhibited data are the means ± SE of no less than three biological replications (n ≥ 3). The data were subjected to a one-way ANOVA according to Duncan’s multiple comparison range test or unpaired two-tailed Student’s t test for the significant differences at *p* = 0.05 with SAS 8.2 and GraphPad Prism 8.0.2, respectively. The main and interactive effects of the solution treatments and longevity of the vase life on the maximum flower diameters are determined by a two-way ANOVA ‘Linear models’ method of the *F*-test subjected to Fisher’s least significant difference (LSD) test with an SAS 8.2 program, where four treatment solutions and the longevity of the vase life of the flowers were regarded as the independent variable, while the maximum flower diameter was regarded as the dependent variable. A map of the study area was drawn with DIVA-GIS7.5. The bar graphs were plotted with GraphPad Prism 8.0.2. The PCA (principal component analysis) was performed using Origin 2022.

## 5. Conclusions

To sum up, the current study demonstrated that a Si addition to the holding solutions significantly improved the postharvest performance of cut peony flowers, as characterized by the extended vase life and well-maintained water status. However, the co-application of Si and a preservative remarkably promoted the postharvest performance and a greater flower diameter, as well as induced an early flowering. In addition, according to the antioxidant capacity and carbohydrate contents obtained in the wilted petals, solely Si-treated stems had reinforced the production of the antioxidant enzymes and diminished the ROS concentration but did not exhibit increased carbohydrate levels compared to those cultivated with the preservative in the holding solutions.

Thus, co-applications of Si and a preservative supplemented to the holding solution was a successful nutritional strategy in improving the postharvest performance of cut peony flowers. A further endeavor appears mandatory to acquire more data on the dynamic changes in the carbohydrate stock, which may confirm the various roles of Si in the postharvest performance of cut flowers.
